# An Unexpected Cause of Extreme Hyperbilirubinemia and Acute Renal Failure: A Case of Weil’s Disease in Poland

**DOI:** 10.7759/cureus.108855

**Published:** 2026-05-14

**Authors:** Martyna Drupka, Krzysztof Pol, Lukasz Holubiuk, Weronika Walecik-Kot

**Affiliations:** 1 Medical School, Medical University of Warsaw, Warsaw, POL; 2 Department of Internal Medicine and Gastroenterology, Masovian Bródno Hospital, Warsaw, POL

**Keywords:** acute kidney injury, acute liver damage, hyperbilirubinemia, jaundice, leptospirosis, rat exposure, thrombocytopenia, weil’s disease

## Abstract

Leptospirosis is a zoonotic disease caused by *Leptospira* spirochetes. It is widespread worldwide, but remains uncommon in European countries. People who come into contact with water, soil, and animals, especially wild rodents, are at risk of infection. The course of the disease is usually mild, but severe cases presenting with multiorgan dysfunction are potentially fatal. When leptospirosis is suspected, rapid treatment is essential for avoiding life-threatening complications. This report discusses the case of a 51-year-old man who was admitted to the hospital with a history of several days of fever, weakness, vomiting, abdominal pain, and jaundice. His condition quickly deteriorated. Initial test results indicated acute liver and kidney injury and thrombocytopenia. Leptospirosis was considered when, upon further questioning, the patient reported the presence of rats in the kitchen where he worked. Serological tests supported the diagnosis of probable leptospirosis. Empirical treatment with ceftriaxone was effective as the patient’s condition improved significantly. This case describes the dynamic course of severe leptospirosis, a disease that is very rare in Poland.

## Introduction

Leptospirosis is a disease caused by bacteria of the genus *Leptospira*. It occurs worldwide, but the highest incidence is observed in warm-climate countries. It is a zoonosis, which means it is transmitted to humans through direct or indirect contact with animals. Infection most often occurs through exposure to infected urine produced by rodents, domestic animals, and livestock [1]. There are two main forms of the disease: mild, with nonspecific symptoms such as weakness, abdominal pain, headache, and muscle pain; and severe, also known as Weil’s disease, with rapid progression, characterized by jaundice, multi-organ damage (particularly liver and kidney injury), and bleeding [1]. Leptospirosis remains rare in European countries, including Poland [2,3]. Due to its low incidence and nonspecific clinical presentation, it may be underrecognized or misdiagnosed, especially in non-endemic areas. This report presents a case of Weil’s disease, the novelty of which lies in unusually severe hyperbilirubinemia and acute kidney injury, as well as the diagnostic challenge posed by its nonspecific presentation in a region where leptospirosis is relatively uncommon.

## Case presentation

A 51-year-old man, a remand prisoner, with no significant past medical history, presented to the hospital with a four-day history of high-grade fever (up to 40 °C), progressive weakness, vomiting, abdominal pain, and shortness of breath. At that time, he was taking paracetamol (up to 2 g per day) and acetylsalicylic acid (up to 1200 mg per day).

The patient denied taking any other medications or consuming wild mushrooms lately. There was no history of recent travel. He had been incarcerated for approximately nine years and reported no alcohol consumption since then. He had his last tattoos done several years ago. He had no chronic illnesses, did not take any long-term medications, and denied any drug allergies. The patient initially attributed his symptoms to food poisoning.

Day 1

Upon admission, the patient was in relatively good general condition, respiratorily and hemodynamically stable, alert, and fully oriented. No meningeal signs were present. Physical examination revealed a blood pressure of 112/84 mmHg, heart rate of 110 beats per minute, peripheral oxygen saturation (SpO_2_) of 99% on room air, and a body temperature of 36.5 °C. The patient was obese, and his skin was jaundiced. Erosions and traces of fresh blood were present on his lips and within the oral cavity. The abdominal examination revealed a soft abdomen with tenderness in the right upper quadrant, without peritoneal signs or palpable masses. Percussion tenderness over the liver was present, and the Goldflam sign was negative bilaterally.

Abdominal ultrasound demonstrated an enlarged liver with increased echogenicity, without focal lesions. Bile ducts were not dilated. The gallbladder was normal, thin-walled, and free of gallstones. Renal ultrasound showed no abnormalities and no signs of obstruction. Chest X-ray revealed no significant pathology.

Initial laboratory findings included notably elevated inflammatory markers with high levels of C-reactive protein (CRP), leukocytosis, neutrophilia, lymphopenia, and thrombocytopenia. Liver function tests showed marked hyperbilirubinemia with total bilirubin of 17.7 mg/dL, elevated levels of aminotransferases: aspartate aminotransferase (AST) and alanine aminotransferase (ALT), gamma-glutamyl transferase (GGT), and alkaline phosphatase (ALP). Renal function was severely impaired with creatinine of 6.27 mg/dL and an estimated glomerular filtration rate (eGFR) of 9 mL/min/1.73 m^2^. Significant hyponatremia and hypokalemia were also noted.

Due to his clinical condition, the patient was treated with intravenous (i.v.) fluid therapy (0.9% sodium chloride and 5% glucose solutions) and loop diuretic administration (torasemide); low-molecular-weight heparin (LMWH) at a prophylactic dose, adjusted to the eGFR; a broad-spectrum antibiotic, ceftriaxone, at a dose of 2 g i.v. twice daily; lactulose and rifaximin for prophylaxis of hepatic encephalopathy; and a prophylactic dose of a proton pump inhibitor (PPI). Electrolyte and fluid imbalances were actively corrected.

Day 2

On the following day, the patient’s condition deteriorated, with blood pressure decreasing to 95/72 mmHg and tachycardia with a heart rate of 112 beats per minute. He remained conscious, oriented, and without signs of hepatic encephalopathy. Physical examination revealed that the abdomen was soft, distended, and non-tender. Bilateral symmetrical edema of the lower legs was observed.

Laboratory tests showed persistent inflammatory activity and progressive hepatorenal dysfunction. Urinalysis revealed cloudy urine with the presence of protein, bilirubin, leukocytes, and erythrocytes (Table 1).

**Table 1:** Urinalysis on day 2 of hospital stay. HPF: high-power field; "-" indicates no reference range.

Parameter	Reference range	Result on day 2
Color	Yellow	Yellow
Clarity	Clear	Cloudy
Specific gravity	1.003-1.030 kg/L	1.015
pH	5-7.5	6
Glucose, qualitative	Negative	Negative
Protein, qualitative	Negative	Positive
Ketones	Negative	Negative
Bilirubin	Negative	Positive
Urobilinogen	0.2-1 EU/dL	0.2
Nitrite	Negative	Negative
Protein	Negative	47.3 mg/dL
Glucose	Negative	Negative
Squamous epithelial cells	Few	Occasional
Dysmorphic erythrocytes	0-3/HPF	3-4/HPF
Leukocytes	0-8/HPF	6-8/HPF
Amorphous urates	-	Fairly numerous

A panel of tests for possible viral etiologies, including hepatitis B virus (HBV), hepatitis C virus (HCV), Epstein-Barr virus (EBV), and cytomegalovirus (CMV), was performed, but no acute infections were detected. Human immunodeficiency virus (HIV) screening was negative, and the alpha-fetoprotein level was normal. Ferritin level was approximately 10 times the upper limit. Blood and urine cultures were also obtained as part of the diagnostic workup. The previously initiated treatment was maintained.

Days 3-4

Over the following days, the liver function tests showed progressive hyperbilirubinemia. Creatinine levels increased to a peak of 7.82 mg/dL, and eGFR decreased to a minimum of 7 mL/min/1.73 m^2^. A peripheral blood smear (Table 2) revealed a neutrophil predominance. Since the CRP level gradually decreased, the selected treatment and antibiotic therapy were continued.

**Table 2:** Peripheral blood smear on day 3 of hospital stay. "-" indicates no reference range.

Parameter	Reference range	Result on day 3
Myelocytes	-	1%
Metamyelocytes	-	1%
Band neutrophils	-	20%
Segmented neutrophils	-	66%
Monocytes	-	6%
Lymphocytes	-	5%
Plasma cells	-	1%

Upon further questioning, the patient reported seeing rats in the prison kitchen where he worked. Given this potential exposure, testing for leptospirosis was ordered.

Days 5-6

On day 6 of hospital stay, total bilirubin rose to a peak of 36.6 mg/dL and direct bilirubin rose to a peak of 26.64 mg/dL, while aminotransferase levels remained relatively moderate. The international normalized ratio (INR) remained within normal limits. D-dimer and lactate dehydrogenase (LDH) levels were markedly elevated. The trend of laboratory parameters throughout hospitalization is summarized in the table below, with the highest and lowest values of the laboratory parameters highlighted (Table 3). Blood and urine culture results were negative.

**Table 3:** Laboratory progression over hospitalization. WBC: white blood cell count; RBC: red blood cell count; MCV: mean corpuscular volume; NEUT%: neutrophil percentage; CRP: C-reactive protein; AST: aspartate aminotransferase; ALT: alanine aminotransferase; ALP: alkaline phosphatase; GGT: gamma-glutamyl transferase; LDH: lactate dehydrogenase; INR: international normalized ratio; eGFR: estimated glomerular filtration rate; AFP: alpha-fetoprotein; "ND": not done; test was not performed on that day. Bolded values indicate the highest or lowest recorded values of the respective parameters during hospitalization.

Parameter	Reference range	Admission	Day 2	Day 4	Day 6	Discharge
WBC	4-10×10^3^/µL	19.36	24.31	27.04	20.4	9.52
RBC	4.6-6.1×10^6^/µL	4.11	3.96	3.76	3.88	3.36
Hemoglobin	13-17.5 g/dL	12.9	12.3	11.8	12	10.5
MCV	79-95 fL	81.5	80.8	79.3	79.1	90.2
Fe	31-144 µg/dL	ND	83	ND	ND	ND
Neutrophils	1.6-6.1×10^3^/µL	17.58	21.27	21.85	15.48	7.14
NEUT%	34-71%	90.8	87.5	80.8	75.9	75
Lymphocytes	1.18-3.74×10^3^/µL	0.5	0.86	0.94	0.68	1.17
Platelets	150-400×10^3^/µL	51	64	119	185	319
CRP	< 5.00 mg/L	126.7	112	67	26.8	4.4
Procalcitonin	< 0.1 ng/mL	ND	ND	3.41	3.17	0.24
Total bilirubin	0.3-1.2 mg/dL	17.7	24.7	ND	36.6	4.7
Direct bilirubin	0.0-0.5 mg/dL	ND	20.42	ND	26.64	ND
AST	0-37 U/L	373	330	ND	193	46
ALT	0-55 U/L	221	223	ND	167	ND
ALP	40-150 U/L	160	158	ND	164	124
GGT	12-64 U/L	141	123	ND	ND	68
LDH	125-243 U/L	ND	718	ND	765	301
INR	0.8-1.2	1.11	1.2	ND	1.04	ND
Fibrinogen	200-400 mg/dL	ND	ND	ND	458	ND
D-dimer	0-500 ng/mL	ND	ND	ND	3019	ND
Creatinine	0.7-1.25 mg/dL	6.27	ND	7.82	6.97	2.8
eGFR	> 90 mL/min/1.73 m^2^	9	ND	7	8	25
Urea	19-44 mg/dL	ND	ND	221	220	ND
Na^+^	137-145 mmol/L	118	114	115	118	131
K^+^	3.5-5.1 mmol/L	3.2	3.8	3.5	4.1	5.6
Ferritin	21.8-274.7 ng/mL	ND	2856.5	ND	ND	ND
AFP	0.89-8.78 ng/mL	ND	< 2.0	ND	ND	ND

Serologic testing using an ELISA assay was positive for anti-*Leptospira* IgM antibodies and negative for anti-*Leptospira* IgG antibodies, while the serum PCR was negative (Table 4). The findings were consistent with the patient’s clinical picture and with probable leptospirosis (Weil’s disease). As further improvement in inflammatory markers was observed in the patient’s laboratory results, the treatment was maintained.

**Table 4:** Results of serologic and infectious investigations. CMV: cytomegalovirus; EBV: Epstein-Barr virus; HIV: human immunodeficiency virus; PCR: polymerase chain reaction.

Test	Result
Hepatitis B surface antigen	Negative
Hepatitis B core antibody	Positive
Hepatitis B surface antibody	Positive
Hepatitis C antibody	Positive
CMV IgM	Negative
CMV IgG	Positive
EBV viral capsid IgM	Negative
EBV viral capsid IgG	Positive
EBV nuclear antigen IgG	Positive
EBV early antigen IgG	Negative
HIV antigen/antibody	Negative
Blood cultures	Negative
Urine cultures	Negative
Anti-*Leptospira* IgM	Positive
Anti-*Leptospira* IgG	Negative
*Leptospira* PCR	Negative

Clinical course and outcome

With the implemented treatment, inflammatory markers gradually decreased, and progressive improvement in renal and hepatic parameters was observed. The patient’s general condition improved accordingly. Although the patient required further monitoring of diuresis and electrolyte levels due to polyuria, the polyuria showed a tendency to be self-limiting.

At the time of discharge, the follow-up laboratory testing revealed moderate normocytic anemia. The patient was transferred for continued care to the prison hospital. Discharge recommendations included continuation of intravenous ceftriaxone at a dose of 2 g twice daily, to complete a minimum total duration of 14 days of therapy; continuation of fluid therapy; continuation of prophylactic LMWH and PPI therapy. Outpatient follow-up in an infectious disease clinic was advised due to a reactive anti-hepatitis C virus (anti-HCV) antibody test result, and further evaluation of anemia was recommended. The necessity of immediate pest control in the prison kitchen was also conveyed to the prison service.

## Discussion

Leptospirosis is caused by *Leptospira* spirochetes. The zoonosis is endemic in tropical and subtropical regions [1]. A wide range of animals serve as reservoirs, including mammals, birds, reptiles, and amphibians. Human infection typically occurs through exposure of mucous membranes or abraded skin to the bodily fluids of infected animals, via animal bites, or through contact with water or soil contaminated with infected urine. Cases of leptospirosis are most often related to occupation, including working in mining, agriculture, sewage systems, veterinary medicine, fisheries, and animal slaughterhouses. Poor sanitary conditions and low socioeconomic status are also associated with an increased risk of exposure to *Leptospira* bacteria [1].

The clinical course of leptospirosis consists of a septic phase associated with bacterial dissemination in the bloodstream, followed by an immune phase related to immunoglobulin production and the presence of spirochetes in host tissues [4]. Based on disease severity, leptospirosis may be categorized into a mild anicteric form and a severe icteric form. The majority of cases present with a mild anicteric form manifesting with nonspecific flu-like symptoms, nausea, abdominal pain, and myalgia. This form is often self-limiting [1]. Approximately 10% of patients develop a severe icteric form, also known as Weil’s disease. It is characterized by jaundice, hemorrhagic diathesis, and multiorgan dysfunction, particularly involving the liver and kidneys [5]. Severe leptospirosis may also progress to complications such as pulmonary hemorrhage, acute respiratory distress syndrome, rhabdomyolysis, and myocarditis [1].

This case illustrates a severe form of Weil’s disease with classic symptoms such as high fever, liver dysfunction with jaundice, acute kidney injury, and thrombocytopenia. It posed a significant diagnostic challenge on several counts.

This patient’s case clearly demonstrates the two-phase nature of leptospirosis. The disease began with several days of high fever with markedly elevated levels of inflammatory markers, followed by progressive hepatorenal damage, as evidenced by rapidly increasing bilirubin and creatinine levels.

Hematological disorders are common in leptospirosis and primarily result from damage to vascular endothelial cells caused by spirochetes [6] and the body’s inflammatory response [4]. In this case, significant leukocytosis, neutrophilia, and lymphopenia were caused by an acute immune response to bacterial infection. Endothelial damage leads to vasculitis, resulting in platelet activation and consumption, which was probably the cause of the patient’s thrombocytopenia [1,6]. Elevated D-dimer and fibrinogen levels indicate inflammatory activation of the coagulation system [4,7].

The patient had traces of blood on the lips, oral cavity erosions, and reported mucosal bleeding. Such symptoms are typical in severe leptospirosis and result from epithelial damage and coagulation disorders [4,8].

The main pathomechanisms leading to liver dysfunction are the destruction of intercellular junctions between hepatocytes caused by leptospires and disturbance of bile secretion by the hepatocellular secretory apparatus [6]. They lead to impaired transport of bile and conjugated bilirubin to the bile ducts, which is reflected by high serum levels of direct and total bilirubin. At the same time, necrotic changes in the liver tissue are relatively minor; therefore, aminotransferase levels are moderately elevated, and INR remains within normal limits. Although hyperbilirubinemia is a typical feature of Weil’s disease, bilirubin levels exceeding 30 mg/dL, as observed in the patient, are rare [9].

Renal dysfunction in leptospirosis is multifactorial. It most often manifests as tubulointerstitial inflammation [6]. The underlying mechanisms include the direct nephrotoxic effect of spirochetes, which primarily colonize the proximal tubules and induce an inflammatory response. This leads to tubular cell apoptosis and impaired function of ion transporters, resulting in increased potassium excretion and, consequently, hypokalemia and polyuria [6]. Other causes include a systemic inflammatory response, microcirculatory disturbances associated with vascular endothelial dysfunction, hemodynamic alterations, hyperbilirubinemia, and rhabdomyolysis. The presentation of kidney damage may range from mild forms of proteinuria and urinary sediment abnormalities to severe forms of acute kidney injury (AKI) [10], as was the case with the patient. Patients with severe icteric leptospirosis and accompanying AKI may require supportive renal replacement therapy, especially in cases with significant renal dysfunction. The need for dialysis has been reported in nearly half of severe cases [11].

The greatest challenge in this case was the differential diagnosis. Given the significant liver involvement, conditions that could have caused such severe abnormalities were considered. Marked jaundice raised suspicion of mechanical cholestasis, but the ultrasound examination did not reveal any signs of gallstones or bile duct dilatation [12]. ALP and GGT levels were elevated, but not to the extent typically seen in mechanical biliary obstruction [12]. Therefore, mechanical cholestasis was ruled out.

The next consideration was acute viral hepatitis. Most commonly, it causes a significant increase in aminotransferase levels, with ALT levels often above 1000 U/L [13]. In contrast, the patient’s aminotransferase levels remained at approximately 200-300 U/L. Testing for HBV, EBV, and CMV indicated past infection, making acute viral hepatitis unlikely. In particular, the HBV serological profile (negative Hepatitis B surface antigen with positive Hepatitis B core antibodies and Hepatitis B surface antibodies) was consistent with resolved infection and did not suggest active viral replication. Therefore, HBV DNA PCR testing was not considered indicated. The patient’s anti-HCV antibody test was reactive, but the overall clinical picture did not suggest acute hepatitis C, so the patient was referred to an infectious diseases clinic for further management.

Acute drug-induced liver injury was also considered, but the patient’s laboratory findings did not indicate the massive hepatocyte necrosis that occurs, for example, in paracetamol overdose. Typically, acute drug-induced liver injury presents with aminotransferase levels exceeding 1000 U/L, impaired coagulation factor synthesis, and increased INR [14]. Contrary to that, in the patient’s case, the INR was within normal limits, and aminotransferase levels did not increase to such high levels. Although the patient reported the use of paracetamol prior to admission, the dosage did not exceed the generally accepted therapeutic range, making severe paracetamol-induced hepatotoxicity less likely clinically. Moreover, the clinical presentation, including marked hyperbilirubinemia with relatively moderate aminotransferase elevation and concomitant acute kidney injury, was more consistent with leptospirosis than with drug-induced liver injury.

Alpha-fetoprotein testing was performed to rule out hepatocellular carcinoma [15]. Since the test results were normal, this cause of hyperbilirubinemia and liver dysfunction was ruled out.

In the context of high ferritin levels, hemochromatosis was considered, but the patient’s clinical picture did not suggest a chronic condition. Given the absence of typical manifestations of hemochromatosis like arthralgia, cardiomyopathy, or diabetes [16], along with normal blood iron levels and reduced hemoglobin concentration, hyperferritinemia was interpreted as a response to acute infection [17].

High values of inflammatory markers and leukocytosis, with a neutrophil predominance, led to the suspicion of bacterial sepsis [7], but the urine and blood cultures detected no growth.

Cases of severe leptospirosis initially mimicking thrombotic microangiopathy have been described in the literature [18]. Therefore, thrombocytopenia combined with acute kidney injury, high LDH values, and the patient’s worsening general condition raised suspicion of diseases such as hemolytic uremic syndrome or thrombotic thrombocytopenic purpura. However, there was no laboratory evidence of hemolysis, and the absence of schistocytes in the blood smear made these diseases unlikely.

In both Europe and Poland, leptospirosis is considered a rare disease [2,3]. Because of this and the lack of obvious occupational exposure or travel history, leptospirosis was not immediately suspected. Only after further investigation did it become apparent that the patient had likely been exposed to rats, which are the most common living reservoirs of leptospires. The non-specific nature of the symptoms and low incidence may be the reason for the difficulty or delay in the diagnosis of leptospirosis in non-endemic countries such as Poland. This case shows how important it is to take a thorough medical history in order to make an accurate diagnosis.

Diagnostic tests used in the diagnosis of leptospirosis can be divided into direct methods (detecting the presence of bacteria or their genetic material) and indirect methods (detecting specific antibodies). Direct methods include culture, polymerase chain reaction (PCR), which allows detection of spirochete DNA in the early phase of the disease, and dark-field microscopy. Because antibodies are not detectable right away, serological testing is recommended after 7 days from symptom onset. These methods include the microscopic agglutination test (MAT) and the enzyme-linked immunosorbent assay (ELISA) [4]. In this case, ELISA-based serological testing was performed, whereas MAT testing was not available. The results demonstrated high titers of IgM antibodies, negative IgG antibodies, and a negative serum PCR result. This finding is consistent with the natural course of leptospirosis, in which molecular detection methods are more useful early in the infection, while serological responses dominate in later stages [4].

The treatment of leptospirosis depends on its severity; some self-limiting cases can be treated symptomatically, but most require antibiotic therapy. In mild cases, the following are administered orally: doxycycline (the drug of choice), ampicillin, amoxicillin, or azithromycin. Severe forms are treated with intravenous antibiotics. The drug of choice is penicillin G. Alternatives include ceftriaxone, cefotaxime, ampicillin, amoxicillin, azithromycin, or doxycycline [19]. In this case, due to the severity of the patient’s clinical condition, the possibility of bacterial sepsis, and the lack of a definitive diagnosis at the time of admission, empirical treatment with a broad-spectrum antibiotic (ceftriaxone) was initiated. A higher dosing regimen was used to ensure adequate coverage in the setting of a suspected severe systemic infection. Although penicillin G is considered the first-line treatment for severe leptospirosis [19], ceftriaxone was selected because the diagnosis had not yet been established and broader empirical antimicrobial coverage was considered more appropriate at that stage. At the same time, ceftriaxone is also recognized as an effective treatment for severe leptospirosis, which is why this therapy was continued in this case. Rapid implementation of empirical antibiotic therapy when leptospirosis is suspected prevents severe complications.

In this patient’s case, appropriate antibiotic therapy and supportive treatment resulted in a gradual improvement of his condition and laboratory results. This demonstrates at least partial reversibility of the damage caused by leptospirosis [8,10].

Several limitations of this case should be acknowledged. The first limitation is that serum paracetamol levels were not measured, which represents a diagnostic limitation and precludes definitive exclusion of paracetamol-related hepatotoxicity. However, the overall clinical presentation was more consistent with leptospirosis. A further limitation of this case is that HCV RNA PCR testing was not performed, which precludes definitive confirmation of active hepatitis C virus infection and limits the ability to fully exclude a concurrent acute viral hepatitis flare. Nevertheless, the clinical presentation did not suggest active HCV infection, making a significant contribution to the patient’s condition unlikely.

## Conclusions

Manifestations of leptospirosis can differ in symptoms and severity. Because it can mimic other infections, its diagnosis might be challenging, especially in non-endemic regions. Therefore, when faced with the combination of jaundice, renal insufficiency, and thrombocytopenia, clinicians should consider leptospirosis in the differential diagnosis, even without a history of overt risk factors. This case also highlights the diagnostic challenges associated with reliance on presumptive serological findings in complex hepatorenal presentations. Despite its rarity, leptospirosis may have a severe and potentially fatal course. That is why it is vital to obtain a detailed environmental history from the patient and initiate empiric treatment as soon as leptospirosis is clinically suspected.
